# Co-opetition Relationships and Evolution of the World Dairy Trade Network: Implications for Policy-Maker Psychology

**DOI:** 10.3389/fpsyg.2020.632465

**Published:** 2021-02-02

**Authors:** Feng Hu, Xun Xi, Yueyue Zhang, Rung-Tai Wu

**Affiliations:** ^1^Global Value Chain Research Center, Zhejiang Gongshang University, Hangzhou, China; ^2^Department of Business Administration, College of Management, University of Providence, Taichung, Taiwan

**Keywords:** dairy trade network, topological structure, dairy product supply, dairy product demand, co-opetition relationship, policy-maker psychology

## Abstract

This study conducted a social network analysis of the evolutionary characteristics of the world dairy trade network based on the overall trade pattern. In addition, the evolution of trade blocs and the co-opetition relationships involving dairy products in major countries were analyzed in terms of supply and demand. The results show that continuous and complex changes have taken place in the world’s dairy trade network since 2001. The number of trade entities in dairy products has stabilized since 2012. At present, approximately 94% of countries (regions) are involved in dairy product trade, such that the world dairy trade network exhibits the small-world effect and scale-free property. The world import pattern for dairy products has changed. While export centers have not changed, import centers have shifted from Europe, America, and East Asia to North America, East Asia, and the Middle East. The world dairy trade network consists of the EU trade bloc headed by Germany, the former Soviet Union–Brazil trade bloc, and the Asia–Australia–America trade bloc. The trade blocs have evolved due to geographical positions, historical cultures, and political relations. In a trade bloc, the diversification of import sources is more prominent in demand countries. European and Asian markets have become the main markets of the major exporters. In this study, the evolutionary characteristics of the world dairy trade network and the co-opetition relationships were analyzed to provide scientific support to inform the development of dairy trade policies. The results can provide technical and psychological support to policy-makers in various countries in their dairy trade decision-making.

## Introduction

Dairy trade, as a key component of international trade, is an important link between areas that are rich and poor in dairy resources. In addition to effectively guaranteeing the supply of dairy products and food safety, dairy trade can also ensure mutual complementarity in the dairy industry. With the increasing demand for dairy products and the continuous promotion of dairy integration processes around the world as well as the gradual increase in trade, the contribution of dairy trade to international trade has gradually increased. In 2017, dairy product trade accounted for 4.39% of the total trade (USD 156.79 billion), playing an important role in international trade. Rabobank predicted that the global dairy supply would stop increasing in 2020 and that dairy supply would be insufficient for different reasons in different countries. These reasons can be summarized as follows: poor economic performance, high milk prices, sluggish retail, geopolitical disputes, and bad weather. Therefore, an in-depth analysis of the world’s dairy trade network may have a profound impact on the world’s dairy trade pattern as well as on the psychology and behavior of decision-makers.

Current studies mainly discuss dairy trade based on a single country (Kurata and Ohe, 2020; Liu et al., 2020) or on two countries (Guo et al., 2020; Mao et al., 2020). In terms of content, literature has mainly focused on the following areas: the competitiveness of dairy trade (Khan et al., 2020), trade potential analysis (Sánchez-López et al., 2020), influencing factors (Bogadóttir, 2020), countermeasures (Zhao et al., 2020), and the impact on industry and market development (Peng and Cox, 2006; Zhang et al., 2020). In terms of methods, dairy trade studies have mainly employed mature models such as constant maturity swap, the technological gap model, trade competitiveness, revealed comparative advantage, international market share, and the Global Trade Analysis Project model (Wen et al., 2010; Ohlan, 2014; Dolin et al., 2020; Meyer et al., 2020; Wijaya et al., 2020).

Generally speaking, existing studies have explored the characteristics of global dairy trade in great depth, but few have addressed its overall status, structural characteristics, and evolution. For example, does the dairy trade network exhibit the small-world effect and power law characteristics? How will the complex game relationships between countries (regions) change under the domination of trade network evolution according to supply and demand powers? Are relationships between supplier countries necessarily competitive? For dairy trade powers, it is particularly urgent to resolve such problems, as clarifying trade network relations can help countries adjust industry policies and promote the sustainable development of the global dairy trade. Focusing on the above problems, this study constructed a global dairy trade network to analyze the network’s structural characteristics in terms of network connectivity and centricity. Based on the time nodes of 2001 and 2017, the evolutionary characteristics of dairy trade blocs and their influencing factors were studied using the complex network community detection algorithm. Finally, the co-competition relationship between supply and demand powers was studied from the perspective of supply and demand to more thoroughly understand the function and structure of the world dairy trade network, thus providing a useful reference for countries (regions) with a high demand for dairy products that are also highly dependent on imports.

On the whole, dairy product trade network analysis can reveal the current international dairy product trade pattern, enabling policy-makers to recognize competitive advantages and disadvantages for their domestic dairy products. This recognition can help those who are formulating policies related to dairy product trade to be more level-headed by reducing psychological pressure and improving their rational decision-making. Of course, our paper serves as a reference and highlights the significance of policy-makers in different countries.

## Methods and Data Headings

### Methods

First, a descriptive analysis of the basic trade conditions was conducted and the trade trend for dairy products was analyzed based on changes in total and specific trade. Second, a social network analysis was carried out. Following Schmitz and Helmberger (1970) and Han and Xu (2020), a social network was adopted to analyze changes in density, average shortest path length, centricity, out-degree and in-degree, closeness centricity, betweenness centricity, and trade blocs to reveal the evolutionary characteristics of the world dairy trade network. The specific study methods are described in the literature by Schmitz and Helmberger (1970) and Han and Xu (2020).

### Subjects and Data

In this study, the countries (regions) participating in global dairy trade were abstracted as nodes, and 60 countries (regions), including China, the United States, and France, were selected as subjects^1^. The duration was set from 2001 to 2017 for the following reasons:

(1) From 2001 to 2017, the import and export volume of dairy products in these countries (regions) accounted for 97% of the total trade, with high representativeness.
(2) In order to minimize the impact of the 2008 financial crisis, the study period was extended 7 years before and 9 years after 2008. This approach of using a longer study period served to minimize the impact of any 1 year while ensuring the quality of data.

A 60 × 60 matrix of the dairy trade network was constructed based on trade flows of the 60 countries (regions), and the characteristics of the network were analyzed by ucinet. In addition, Gephi was used to visualize the network. The types of dairy products included in this study were unconcentrated milk and cream, solid milk and cream, yoghurt, whey and modified whey, butter, and cheese, with the corresponding customs HS codes 0401–0406.

**FIGURE 1:**
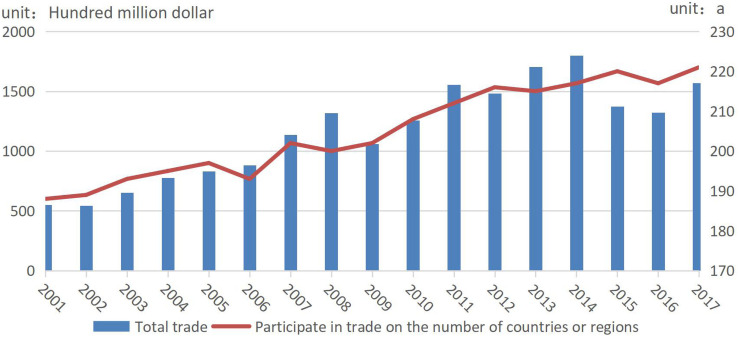
Evolution of total trade of global dairy and participants from 2001 to 2017.

As shown in Figure 1, the total trade of dairy products was only USD 55.104 billion in 2001 and then reached USD 179.878 billion in 2014. It gradually declined in 2014 before dropping sharply in 2015 due to the impact of dairy import construction in China and import bans in Russia (Zhong et al., 2014). In addition, increasing trade and entities in the world dairy market would inevitably change total dairy trade. In 2001, only 188 countries (regions) participated in global dairy trade, while in 2010, the number exceeded 200. In 2012, approximately 94% of countries (regions) participated in global dairy trade, and the number of participating countries (regions) was approximately 215.

**FIGURE 2:**
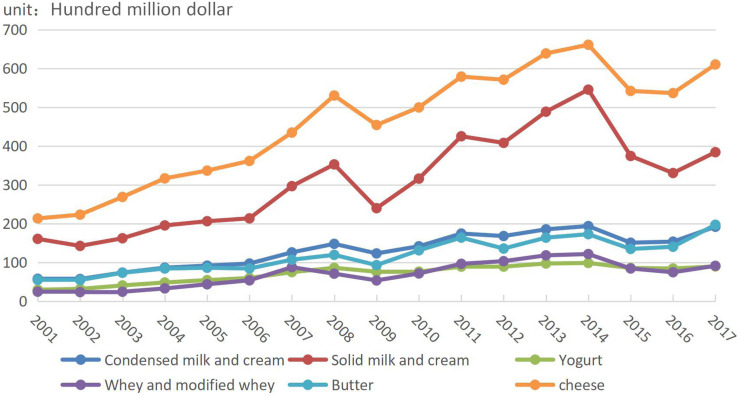
Evolution of types of global dairy trade from 2001 to 2017.

Dairy trade can be divided into three stages according to volume changes in various types of dairy trade (Figure 2). In Stage 1 (2001–2007), total trade and the trade of all types of dairy products gradually increased, with the trade of cheese being slightly higher than that of solid milk and cream and the trade and increment of whey and modified whey being the lowest. In Stage 2 (2008–2013), the trade of unconcentrated milk and cream, yoghurt, whey and modified whey, and butter maintained relatively stable growth while the trade of cheese, solid milk, and cream fluctuated like a “roller coaster,” while total trade also fluctuated. In Stage 3 (2014–2017), the trade of unconcentrated milk and cream, yoghurt, whey and modified whey, and butter continued to grow. Meanwhile, the trade of cheese, solid milk, and cream dropped sharply from 2014 to 2016 and then rose again in 2017.

The complexity of the network increased, exhibiting the small-world network effect.

## Evolution of Connectivity and Node Centricity in the World Dairy Trade Network

### The Complexity of the Network Increased, Exhibiting the Small-World Network Effect

**FIGURE 3:**
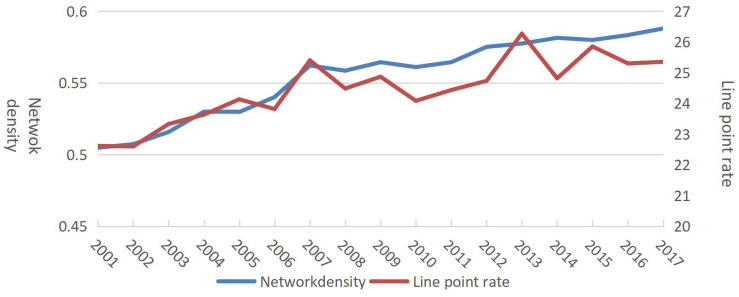
Evolution of network density and line point rate from 2001 to 2017.

First, the trade linkage between countries (regions), network density, complexity, and network line point rate were directly proportional. The changes in network density showed that the density of the dairy trade network increased year by year, with a minimum value of 0.50 in 2001 and a maximum value of 0.59 in 2017. From 2001 to 2017, the network density fluctuated but gradually increased. After 2012, the network density stabilized between 0.57 and 0.58. The changes in the line point rate showed a minimum value of 22.60 in 2002, with it gradually increasing in later years. A comprehensive comparison of network density and line point rate showed that both indicators had basically the same trend (increase) after 2001 (Figure 3), indicating that dairy trade relationships was becoming more complicated as network connectivity gradually improved.

**FIGURE 4:**
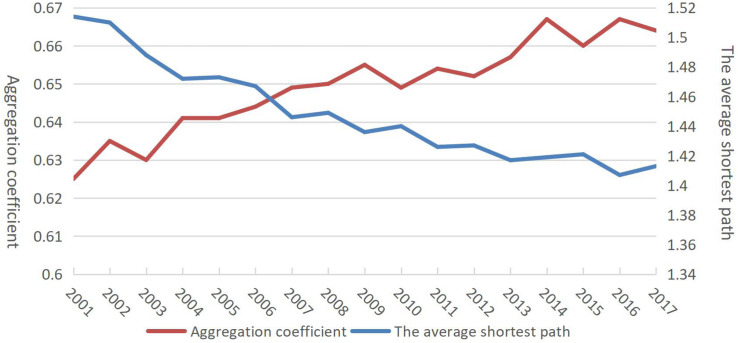
Evolution of aggregation coefficient and average shortest path from 2001 to 2017.

Second, the evolution trend of the aggregation coefficient and the length of the average shortest path (Figure 4) showed that, although the two indicators changed in opposite directions, the aggregation of the network became tighter after 2010. In particular, it can be observed that the aggregation coefficient was 0.63 in 2001 and 0.67 in 2014 before dropping slightly in 2015.

Third, under normal circumstances, a higher aggregation coefficient and shorter average shortest path length may suggest the small-world effect in the network. The longest average shortest path length was 1.44 and highest aggregation coefficient was about 0.62, indicating that the dairy trade network exhibited the small-world effect.

For policy-makers, the advantage of a complex and relatively concentrated dairy product trade network is that it enables them to concentrate on formulating more targeted import and export policies. However, the disadvantages are also relatively obvious. Affected by complex cooperation methods and small-scale cooperation, decision-makers need to be more cautious when facing collaborators, which covertly increases the psychological burden.

### The Network Showed the Scale-Free Property, Changing the Import Pattern

**FIGURE 5:**
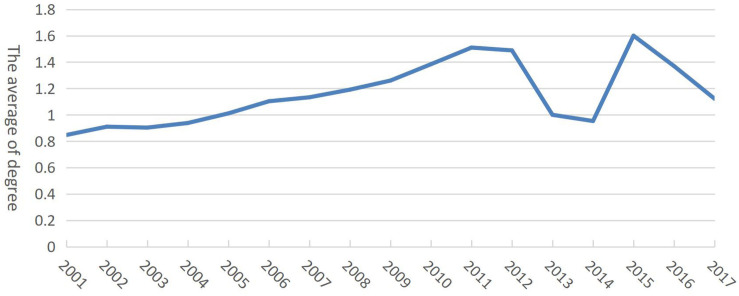
Evolution of the average of degree of global dairy products from 2001 to 2017.

**FIGURE 6:**
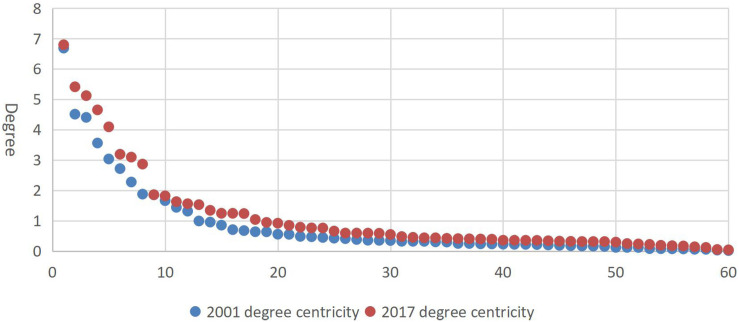
Evolution of degree of global dairy products from 2001 to 2017.

Centricity, as an important indicator of the role of nodes in the network, can reflect the relative importance of countries (regions) in the dairy trade network. The time series showed that the average degree was only 0.85 in 2001 but reached 1.60 in 2015, increasing by two times (Figure 5). Although centricity fluctuated greatly after 2012, the changes showed that the impact and role of countries (regions) in the world dairy trade network were gradually increasing. As shown in Figure 6, the degree curve of the world dairy trade network conformed to the characteristics of the power-law distribution in 2001 and 2017, indicating that the dairy trade network was a scale-free network. Since the degree curve of 2001 was under that of 2017, the scale-free property of the dairy trade network gradually strengthened over time.

In the trade network, the weighted degree can represent the diversification of import and export of dairy products and the distribution diversification of import and export regions (Table 1). Over time, the top 10 countries (regions) with respect to weighted degree, out-degree, and in-degree changed greatly. In 2001, the top three countries for out-degree were Germany, France, and the Netherlands, and the top three countries for in-degree were Germany, Italy, and France. Meanwhile in 2017, the corresponding countries were Germany and the Netherlands (in-degree) and Germany, mainland China, and the Netherlands (out-degree). It should be noted that mainland China did not reach the top 10 with respect to weighted degree, out-degree, and in-degree before 2017, but in 2017, it ranked sixth in average degree and second for in-degree. The geographical distribution characteristics of the top 10 countries (regions) for weighted degree, out-degree, and in-degree in 2001 and 2017 showed that the import pattern of dairy products changed with the diversification of import and export countries (regions) as well as the diversification of products. The import focus of dairy products was transferred from Europe, America, and East Asia to North America, East Asia, and the Middle East, while the export focus did not change greatly. Policy-makers in various countries should adjust their psychological goals to correspond to this evolution of the trade network center to minimize inaccurate decision-making that arises from psychological expectations being too high or low.

**TABLE 1:** Top 10 countries with respect to weighted degree, in-degree, and out-degree.

**2001**					
**Country**	**Degree centricity**	**Country**	**Out-degree**	**Country**	**In-degree**
Germany	6.69	Germany	5.34	Germany	3.96
France	4.51	France	4.18	Italy	3.14
Netherlands	4.40	Netherlands	3.93	France	2.58
Italy	3.56	New Zealand	3.02	Netherlands	2.36
New Zealand	3.03	Belgium	1.98	Belgium	2.36
Belgium	2.72	Australia	1.68	United Kingdom	2.11
United Kingdom	2.27	Denmark	1.64	Spain	1.32
United States	1.87	Ireland	1.30	United States	1.24
Australia	1.85	Italy	1.26	Japan	0.98
Denmark	1.66	United Kingdom	0.94	Mexico	0.95

**2017**					

Germany	6.80	Germany	5.48	Germany	4.59
Netherlands	5.41	Netherlands	5.09	Mainland China	3.18
New Zealand	5.12	New Zealand	5.04	Netherlands	2.57
France	4.65	France	3.86	France	2.55
United States	4.09	Belgium	2.06	Belgium	2.29
Mainland China	3.19	Italy	2.05	Italy	2.29
Italy	3.09	United States	2.04	United States	2.09
Belgium	2.86	Ireland	1.55	Saudi Arabia	1.37
Saudi Arabia	1.86	Denmark	1.53	Sweden	1.16
Ireland	1.82	Belarus	1.22	Uruguay	1.10

### The Network Had a “Point-to-Point” Model, Manifesting as Dependence and Competition Between Countries (Regions)

Closeness centrality refers to the level at which a node is not controlled by the sending and receiving of another node, while betweenness centrality refers to the control level of resources by node countries (regions) in the network. These two indicators can reflect the pattern of dairy trade to a large extent. From 2001 to 2017, despite fluctuations, the closeness centrality steadily increased. The results in 2001 and 2017 showed that the top 10 countries (regions) in 2001 were Denmark, France, the Netherlands, Germany, Italy, the United Kingdom, the United States, Canada, Belgium, and Spain, while in 2017, they were France, Germany, Italy, the Netherlands, Spain, Denmark, Poland, the United Kingdom, the United States, and Slovakia (Table 2). It was found that more than half of the above countries (regions) were listed in the top 10 in-degree or out-degree 10 countries (regions), demonstrating the “point-to-point” characteristic of the trade mode in the world dairy trade network.

**TABLE 2:** Distribution of top 10 countries with respect to closeness centrality and betweenness centrality.

**2001**				**2017**			
**Country**	**Closeness centrality**	**Country**	**Betweenness centrality**	**Country**	**Closeness centrality**	**Country**	**Betweenness centrality**
Denmark	100.00	Denmark	1.59	France	100.00	France	0.81
France	100.00	France	1.59	Germany	100.00	Germany	0.81
Netherlands	100.00	Netherlands	1.59	Italy	100.00	Italy	0.81
Germany	98.33	Germany	1.44	Netherlands	100.00	Netherlands	0.81
Italy	96.72	Italy	1.33	Spain	100.00	Spain	0.81
United Kingdom	96.72	United Kingdom	1.33	Denmark	98.33	United States	0.80
United States	96.72	Spain	1.25	Poland	98.33	United Kingdom	0.77
Canada	95.16	Belgium	1.23	United Kingdom	98.33	Denmark	0.74
Belgium	93.65	United States	1.21	United States	98.33	Poland	0.74
Spain	93.65	Switzerland	1.15	Slovakia	95.16	Switzerland	0.69

As shown in Table 2, the betweenness centrality was maintained between 0.38 and 0.40. The sizing analysis on betweenness centrality in 2001 showed that the top 10 countries (regions) were Denmark, France, the Netherlands, Germany, Italy, the United Kingdom, Spain, Belgium, the United States, and Switzerland. Meanwhile, in 2017, they were France, Germany, Italy, the Netherlands, Spain, the United States, the United Kingdom, Denmark, Poland, and Switzerland. The comparison of betweenness centrality and out-degree showed that six countries (Germany, the Netherlands, France, Italy, the United States, and Denmark) were still included in the top 10 countries (regions) in 2017, showing that the control strength of the relationship between dairy products directly corresponded to dependence between the exporting countries (regions) and their vulnerability to one another.

In general, decision-makers should focus on strengthening interaction and communication with partners outside the network in a strong control trade network. Conversely, in a weak control trade network, they should emphasize strengthening interaction and communication with partners inside the network.

## Bloc Characteristics of Evolution of the Dairy Trade Network and Co-Competition Relationships Between Supply and Demand Powers

### Bloc Detection of Evolution of the Dairy Trade Network

**FIGURE 7:**
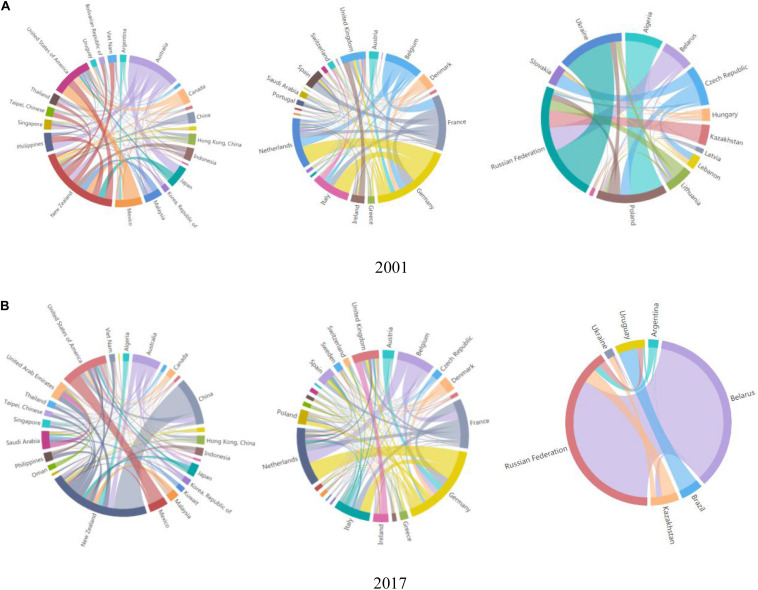
Network structure of world dairy trade blocs. **(A)** 2001, **(B)** 2017.

Under normal circumstances in dairy trade, the path dependence of countries (regions) would promote the formation of trade blocs. The characteristics, trade connections, and scale of dairy trade blocs (Figure 7) showed that three trade blocs coalesced in 2001. First, there was the trade bloc consisting of European and Middle Eastern countries (regions), with Germany as the core node and the Netherlands and France as important nodes. These countries had relatively close trade linkages and a large scale of trade with the other countries (regions) in the bloc. Second, there was the trade bloc of the former Soviet Union countries (led by Russia), with the Russian Federation as the absolute core node and Poland and Ukraine as the important nodes. Lastly, there was the Asia–Australia–America trade bloc, which had relatively close internal connections, with New Zealand and Australia as the core nodes and the United States and Japan as the important nodes.

In 2017, there were still three trade blocs but with an obvious overlapping trend. The trade bloc for the former Soviet Union countries and that of the European countries became one bloc in 2001. This was because in countries (regions) such as Russia, external dependence on dairy products increased gradually, and these countries relied on dairy products from European countries (regions). In this trade bloc, the importance of the Netherlands increased, second only to Germany. Moreover, the original Asia–Australia trade bloc expanded to include Middle Eastern countries (regions). Meanwhile, the importance of China and the United States was lower than that of New Zealand, and the status of Australia declined. Third, the trade bloc of the former Soviet Union countries started shrinking and formed a new bloc with South American countries, with the Russian Federation remaining as the absolute core node of the bloc.

The variation trend in trade blocs from 2001 to 2017 showed that geographical location played an important and fundamental role. For example, internal relations between East Asian and South Asian countries have always been close. In addition, historical and cultural factors are key factors in the formation of a trade bloc. As another example, although the Soviet Union collapsed, the former Soviet Union countries still had close relationships with European countries under the guidance of Russia.

### Import Sources of Major Supply and Demand Powers Were More Diversified From the Perspective of Cooperation

Regarding the number of import sources, Russia had the least (two sources) among all the dairy demand powers. Belgium and Netherlands also had relatively fewer import sources, with three and four, respectively. Italy, mainland China, and France had five import sources, and Spain had six import sources. Germany, the United Kingdom, and the United States had seven import sources, the most among all countries. The import and export concentration rates of the top 10 supply powers showed that the export concentration of the four countries reached 80%, while that of the other six countries was 70–80%. In comparison, only two of the top 10 demand powers had import concentrations above 80%, while five countries had import concentrations of 40–80% and three countries had import concentrations less than 40%, with an average import concentration of 55.4% and average export concentration of 79.78%. In addition, dairy product supply countries had 2.4 exporting countries (regions), while demand countries had 5.1 source countries (regions). From this, it is evident that the import sources of demand power were more diversified than the supply sources of exporting countries. The import concentration rate was far lower than that in the supply countries, further confirming the importance of a diversified development strategy in dairy importing countries.

### A Competitive Relationship Between Supply Countries Did Not Necessarily Exist

In 2017, the export powers for dairy products were New Zealand, Germany, the Netherlands, France, the United States, Belgium, Italy, Denmark, Ireland, and Belarus. As for Belarus, 94.91% of its dairy products were exported to the Russian Federation, and regarding Denmark, 49.83% of its dairy products were exported to three major targets. The Netherlands and New Zealand had three major export targets, with export concentration rates of 59.48 and 50.97%, respectively. Belgium had five export targets, with an export concentration rate of 76.10%. France, Germany, Ireland, and the United States had six export targets each, with Ireland and the United States being more diversified in exports having export concentration rates of 78.16 and 74.04%, respectively.

The competitive relationship between major supply powers for dairy products may rest on export targets. Based on experience, supply powers would have a competitive relationship if they had the same export direction. However, in actual situations, due to differences in export targets and directions, competitive relationships could not be generalized. For example, New Zealand and the Netherlands had different export directions and targets. New Zealand mainly exported to Australia, mainland China, Malaysia, and the United Arab Emirates, while the Netherlands mainly exported to Belgium, France, Germany, and Hong Kong. Therefore, a non-competitive relationship existed between New Zealand and the Netherlands. Meanwhile, Belgium and France engaged in large-scale dairy trade with Germany, Italy, and the United Arab Emirates; thus, there was a competitive relationship between them. In sum, a competitive relationship did not necessarily exist between dairy supply countries. In general, competitive relationships existed among Belgium, France, and Germany, which mainly competed with each other in Europe and Asia.

## Conclusion and Policy Implication

Since 2001, the world dairy trade network has been subject to continuous and complex changes. After 2012, the number of entities in dairy trade stabilized, and presently, approximately 94% of countries (regions) participate in dairy trade. The dairy trade network exhibits the typical small-world effect and scale-free property, which may become more obvious over time. The import focus of dairy products has shifted to North America, East Asia, and the Middle East from Europe, South America, and East Asia, while the export focus remains unchanged. The trade model of the world dairy trade network shows a “point-to-point” characteristic. The control strength of trading countries depends on their mutual dependence and vulnerability. In dairy trade, a country (region) generally has a certain path dependence, and a trade bloc is the embodiment of such dependence from the perspective of colony characters. At present, there are three trade blocs in the world dairy trade network: the Asia–Australia–America trade bloc, the former Soviet Union–Brazil trade bloc, and the EU trade bloc, which is led by Germany. The exchange between these blocs has gradually deepened, and the blocs have evolved based on their geographical locations, historical cultures, and political relations. In a trade bloc, demand powers are more diversified in import sources than are supply powers. A competitive relationship does not necessarily exist between supply powers. In general, competitive relationships exists among Belgium, France, and Germany, which compete with each other mainly in Europe and Asia.

First, there is mutual dependency and vulnerability between supply and demand countries. The corresponding countries (regions) can explore potential import and export markets based on trade agreements, further adjusting the market and product structures for dairy products. Second, the demand and import powers should continue to deepen and stabilize trade relationships with supply countries and strengthen the macro-control of dairy products by meeting demand for these products. Third, large producing countries should strengthen the competitiveness of their dairy industries by continuously improving the quality of dairy products, supporting export of dairy companies, expanding export markets, and accelerating the promotion of multilateral trade cooperation mechanisms.

In relation to existing studies, network analysis was adopted in this study to discuss dairy trade networks between countries. In addition, the structures of networks were also analyzed in terms of network connectivity and centrality. Our findings can provide technical and psychological support to various countries in their dairy trade decision-making processes. In addition, the dairy product trade network analysis reveals current patterns in international dairy product trade, the understanding of which can enable policy-makers to recognize competitive advantages and disadvantages of their domestic dairy products. This understanding can help these policy-makers to be more level-headed when formulating policies related to dairy product trade by reducing psychological pressure and improving rational decision-making abilities. However, the impact of the current dairy trade network on the internal dairy industry development in each participating country (region) was not discussed in this study due to data accessibility limitations. Subsequent studies should focus on this topic.

## Data Availability Statement

The original contributions presented in the study are included in the article/supplementary material, further inquiries can be directed to the corresponding author.

## Author Contributions

All authors undertook research, writing, review tasks throughout this study, read, and agreed to the published version of the manuscript.

## Conflict of Interest

The authors declare that the research was conducted in the absence of any commercial or financial relationships that could be construed as a potential conflict of interest.

## Footnotes

1 Germany, Netherlands, France, New Zealand, Italy, Belgium, United Kingdom, United States, Denmark, Spain, Ireland, Australia, China, Saudi Arabia, Poland, Russian Federation, Austria, Mexico, Belarus, Greece, Japan, Hong Kong, Czech Republic, Sweden, Algeria, Switzerland, Singapore, United Arab Emirates, Malaysia, Indonesia, Portugal, Argentina, Finland, Philippines, Egypt, Luxembourg, Lithuania, Thailand, Canada, Nigeria, Oman, Slovakia, North Korea, Hungary, Iran, Brazil, Vietnam, Venezuela, Uruguay, Ukraine, Kuwait, Taiwan, Kuwait, Romania, Chile, Lebanon, Latvia, Slovenia, Bahrain, and Kazakhstan.
